# 5,11,17,23-Tetra­bromo-25,26,27,28-tetra­kis(4-tolyl­sulfon­yloxy)-2,8,14,20-tetra­thia­calix[4]arene dichloro­methane solvate

**DOI:** 10.1107/S1600536810009554

**Published:** 2010-03-20

**Authors:** Yue-Feng Chen, Yang Liu, Jian-Ping Ma, Dian-Shun Guo

**Affiliations:** a Department of Chemistry, Shandong Normal University, Jinan 250014, People’s Republic of China

## Abstract

In the crystal structure of the title compound, C_52_H_36_Br_4_O_12_S_8_·CH_2_Cl_2_, the thia­calix[4]arene unit adopts a 1,3-alternate conformation with an intra­molecular C—H⋯O hydrogen bond and four C—H⋯π inter­actions, with the four 4-MeC_6_H_4_SO_3_ groups located alternately above and below the virtual plane (*R*) defined by the four bridging S atoms. The benzene ring of each 4-MeC_6_H_4_SO_3_ unit is nearly perpendicular to one of the two neighboring phenol rings with inter­planar angles varying from 72.97 (13) to 78.70 (13)°, while the dihedral angles between the plane (*R*) and the phenol rings range from 83.04 (7) to 84.30 (9)°. In the supra­molecular structure, a solvent-bridged dimer composed of two main mol­ecules is formed by four inter­molecular C—H⋯O hydrogen bonds and locally creates an *R*_4_^4^(26) motif. Such dimers associate further into chains by inter­dimer C—Cl⋯O short contacts [Cl⋯O 3.182 (5) Å]. Finally, these chains are linked into a two-dimensional network by a combination of inter­chain C—Br⋯O inter­actions [Br⋯O = 3.183 (3) and 2.966 (4) Å] as well as C—H⋯O hydrogen bonds.

## Related literature

For general background to the chemistry of thia­calix[4]arenes, see: Kumagai *et al.* (1997 ▶); Shokova & Kovalev (2003 ▶); Lhoták (2004 ▶); Morohashi *et al.* (2006 ▶); Guo *et al.* (2007 ▶). For the synthesis and related structures, see: Lhoták *et al.* (2001 ▶); Kasyan *et al.* (2003 ▶); Xu *et al.* (2008 ▶). For C—H⋯π inter­actions, see: Tsuzuki *et al.* (2000 ▶). For hydrogen-bond motifs, see: Bernstein *et al.* (1995 ▶). For C—*X*⋯O (*X* = Cl, Br) short contacts, see: Lommerse *et al.* (1996 ▶); Metrangolo & Resnati (2001 ▶). For atomic radii, see: Bondi (1964 ▶).
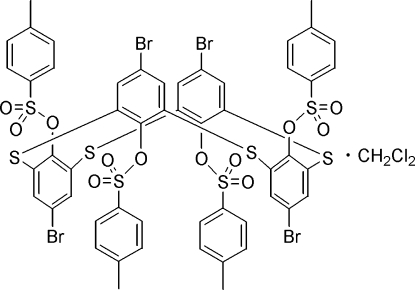


## Experimental

### 

#### Crystal data

C_52_H_36_Br_4_O_12_S_8_·CH_2_Cl_2_
*M* *_r_* = 1513.85
Monoclinic, 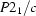

*a* = 12.6945 (14) Å
*b* = 20.793 (2) Å
*c* = 23.190 (3) Å
β = 103.350 (2)°
*V* = 5955.8 (12) Å^3^
*Z* = 4
Mo *K*α radiation
μ = 3.13 mm^−1^
*T* = 173 K
0.30 × 0.21 × 0.12 mm

#### Data collection

Bruker SMART CCD area-detector diffractometer
Absorption correction: multi-scan (*SADABS*; Bruker, 1999 ▶) *T*_min_ = 0.453, *T*_max_ = 0.705
30976 measured reflections
11092 independent reflections
7807 reflections with *I* > 2σ(*I*)
*R*_int_ = 0.039

#### Refinement

*R*[*F* ^2^ > 2σ(*F* ^2^)] = 0.040
*wR*(*F* ^2^) = 0.107
*S* = 1.06
11092 reflections
715 parameters
6 restraints
H-atom parameters constrained
Δρ_max_ = 0.86 e Å^−3^
Δρ_min_ = −0.68 e Å^−3^

### 

Data collection: *SMART* (Bruker, 1999 ▶); cell refinement: *SAINT* (Bruker, 1999 ▶); data reduction: *SAINT*; program(s) used to solve structure: *SHELXS97* (Sheldrick, 2008 ▶); program(s) used to refine structure: *SHELXL97* (Sheldrick, 2008 ▶); molecular graphics: *SHELXTL* (Sheldrick, 2008 ▶); software used to prepare material for publication: *SHELXTL*.

## Supplementary Material

Crystal structure: contains datablocks I, global. DOI: 10.1107/S1600536810009554/bg2335sup1.cif

Structure factors: contains datablocks I. DOI: 10.1107/S1600536810009554/bg2335Isup2.hkl

Additional supplementary materials:  crystallographic information; 3D view; checkCIF report

## Figures and Tables

**Table 1:** Hydrogen-bond geometry (Å, °) *C*g1, *C*g2, *C*g3 and *C*g4 are the centroids of the C14–C19, C1–C6, C51–C56 and C33–C38 rings, respectively.

*D*—H⋯*A*	*D*—H	H⋯*A*	*D*⋯*A*	*D*—H⋯*A*
C12—H12⋯O8	0.95	2.51	3.303 (5)	141
C53—H53*A*⋯O5^i^	0.99	2.38	3.175 (5)	137
C53—H53*B*⋯O4^ii^	0.99	2.37	3.249 (5)	147
C17—H17⋯O2^iii^	0.95	2.38	3.266 (5)	155
C10—H10⋯*Cg*1	0.95	2.75	3.698 (1)	178
C23—H23⋯*Cg*2	0.95	2.65	3.602 (1)	176
C30—H30⋯*Cg*3	0.95	2.80	3.742 (1)	174
C41—H41⋯*Cg*4	0.95	2.85	3.794 (1)	172

Symmetry codes: (i) 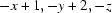; (ii) 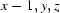; (iii) 
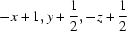
.

**Fig. 1.**
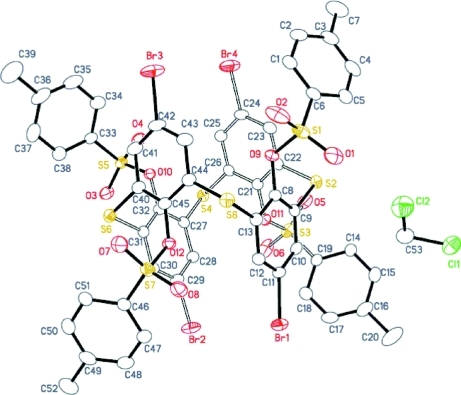
The molecular structure of the title compound with displacement ellipsoids drawn at the 30% probability level for non-H atoms.

**Fig. 2.**
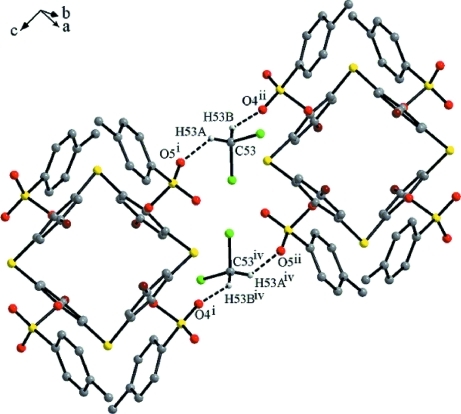
A view of the hydrogen-bonded dimer of the title compound approximatively along the b axis with an R44(26) motif. Some hydrogen atoms are omitted for clarity. [Symmetry codes: (i) -x + 1, -y + 2, -z; (ii) x - 1, y, z; (iv) -x, -y + 2, -z].

**Fig. 3.**
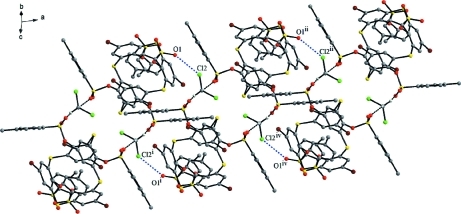
A view of the one-dimensional chain of the dimers approximatively along the b axis. Some hydrogen atoms are omitted for clarity. [Symmetry codes: (i) -x + 1, -y + 2, -z; (ii) x - 1, y, z; (iv) -x, -y + 2, -z].

## supplementary crystallographic information

### Comment

Thiacalix[4]arenes have attracted considerable interest in recent years as versatile scaffolds for highly organized receptors (Kumagai *et al.*, 1997; Shokova & Kovalev, 2003; Lhoták, 2004; Morohashi *et al.*, 2006; Guo *et al.*, 2007). Moreover, they are able to undergo electrophilic bromination at the upper rim and generate the corresponding dibromo or tetrabromo thiacalix[4]arene derivatives (Lhoták *et al.*, 2001; Kasyan *et al.*, 2003; Xu *et al.*, 2008), which can be further applied to construct more elaborate receptors as well as novel supramolecular systems. We report here the crystal structure of a new tetrabromo thiacalix[4]arene derivative, 5,11,17,23-tetrabromo-25,26,27,28-tetrakis(4-toluenesulfonyloxy)-2,8,14,20-tetrathiacalix[4]arene dichloromethylene solvate.

In the crystal structure of the title compound, as shown in Fig. 1, the thiacalix[4]arene platform adopts a 1,3-alternate conformation in which the four 4-MeC_6_H_4_SO_3_ groups are located alternately above and below the virtual plane (*R*) defined by four bridging S atoms. The dihedral angles between the plane (*R*) and the phenol rings vary from 83.04 (7) to 84.30 (9)°. Such an arrangement is different from that of its analogue where four OCH_2_CO_2_Me moieties replace the four 4-MeC_6_H_4_SO_3_ units, possessing a partial cone conformation (Xu *et al.*, 2008). Interestingly, four C—H···π interactions as well as one intramolecular C12—H12···O8 hydrogen bond (Table 1) in each thiacalix[4]arene molecule result in the benzene ring of each 4-MeC_6_H_4_SO_3_ group leaning aslant and being nearly perpendicular to one of both neighboring phenolic rings, with dihedral angles ranging from 72.97 (13) to 78.70 (13)°. The distances of H10···*C*g1, H23···*C*g2, H30···*C*g3 and H41···*C*g4 (*C*g1, *C*g2, *C*g3 and *C*g4 are the centroids of the rings C14–C19, C1–C6, C51–C56 and C33–C38, respectively) are 2.749 (1), 2.654 (1), 2.795 (1) and 2.851 (1) Å , respectively, which are consistent with the calculations for such interactions conducted by Tsuzuki *et al.* (2000).

In the supramolecular structure, non-classical C—H···O hydrogen bonds (Table 1) and C—X···O (X = Cl, Br) short contacts (Lommerse *et al. *1996; Metrangolo & Resnati, 2001) are observed. Pairs of the thiacalix[4]arene molecules are bridged by two dichloromethylene solvents with four intermolecular C—H···O hydrogen bonds and form a solvent-bridged centrosymmetric dimer (Fig. 2), locally creating an *R*_4_^4^(26) motif (Bernstein *et al.*, 1995) from atoms C53—H53*A* and C53—H53*B* at (x, y, z) and (-x, -y + 2, -z), respectively, acting as hydrogen-bond donors to atoms O5 at (-x + 1, -y +2, -z) and (x - 1, y, z), and O4 at (x - 1, y, z) and (-x + 1, -y +2, -z). Such dimers associate further into one-dimensional chains (Fig. 3), approximatively along the crystallographic *b* axis, by interdimer C53—Cl2···O1 short contacts (Metrangolo *et al. *2001). Finally, these chains are linked into a two-dimensional network by a combination of interchain C11—Br1···O2 and C29—Br2···O1 interactions as well as C17—H17···O2 hydrogen bonds (Table 1). The separations between Cl2 and O1, Br1 at (-x + 1, y - 1/2, -z + 1/2) and O2, and Br2 at (-x + 1, y - 1/2, -z + 1/2) and O1 are 3.182 (5), 3.183 (3) and 2.966 (4) Å, respectively, which are shorter than the sum of the van derWaals radii of the halogen and O atoms (O = 1.52 Å, Br = 1.85 Å, Cl = 1.75 Å; Bondi, 1964).

### Experimental

A suspension of *p*-tetrabromothiacalix[4]arene (0.250 g, 0.308 mmol), anhydrous Cs_2_CO_3 _(0.610 g, 1.846 mmol) and *p*-toluenesulfonyl chloride (0.620 g, 3.078 mmol) in dry acetonitrile (15 ml) was refluxed for 20 h and cooled to room temperature. After removal of the volatile under reduced pressure, the residue was neutralized with 5% aqueous HCl and extracted with CH_2_Cl_2_. The organic layer was washed with saturated sodium hydrogen carbonate and brine, and dried over anhydrous MgSO_4_. After removal of the solvent under reduced pressure, the residue was purified by flash column chromatography (silica gel, ethyl acetate/hexane = 1:5, *R*_f_ = 0.5) to give the title compound in 58 % yield as a white solid. ^1^H NMR (300 MHz, CDCl_3_): *δ*8.00 (d, 8H, *J* = 8.03 Hz), 7.51 (d, 8H, *J* = 8.00 Hz), 7.28 (s, 8H), 2.55 (s, 12H). Single crystals suitable for X-ray diffraction analysis were obtained by slow evaporation of a solution in CH_3_OH and CH_2_Cl_2_ at 273 K.

### Refinement

All non-hydrogen atoms were refined with anisotropic displacement parameters. Hydrogen atoms attached to refined atoms were placed in geometrically idealized positions and refined using a riding model, with C—H = 0.93, 0.98 and 0.97 Å for aromatic, methylene and methyl H, respectively, and Uiso(H) = 1.5Ueq(C) for methyl H, and Uiso(H) = 1.2Ueq(C) for all other H atoms.

### Crystal data

**Table:**

C_52_H_36_Br_4_O_12_S_8_·CH_2_Cl_2_	*F*(000) = 3016
*M**_r_* = 1513.85	*D*_x_ = 1.688 Mg m^−^^3^
Monoclinic, *P*2_1_/*c*	Mo *K*α radiation, λ = 0.71073 Å
Hall symbol: -P 2ybc	Cell parameters from 7685 reflections
*a* = 12.6945 (14) Å	θ = 2.4–26.5°
*b* = 20.793 (2) Å	µ = 3.13 mm^−^^1^
*c* = 23.190 (3) Å	*T* = 173 K
β = 103.350 (2)°	Block, colourless
*V* = 5955.8 (12) Å^3^	0.30 × 0.21 × 0.12 mm
*Z* = 4	

### Data collection

**Table:**

Bruker SMART CCD area-detector diffractometer	11092 independent reflections
Radiation source: fine-focus sealed tube	7807 reflections with *I* > 2σ(*I*)
graphite	*R*_int_ = 0.039
phi and ω scans	θ_max_ = 25.5°, θ_min_ = 1.7°
Absorption correction: multi-scan (*SADABS*; Bruker, 1999)	*h* = −10→15
*T*_min_ = 0.453, *T*_max_ = 0.705	*k* = −25→25
30976 measured reflections	*l* = −27→28

### Refinement

**Table:**

Refinement on *F*^2^	Primary atom site location: structure-invariant direct methods
Least-squares matrix: full	Secondary atom site location: difference Fourier map
*R*[*F*^2^ > 2σ(*F*^2^)] = 0.040	Hydrogen site location: inferred from neighbouring sites
*wR*(*F*^2^) = 0.107	H-atom parameters constrained
*S* = 1.06	*w* = 1/[σ^2^(*F*_o_^2^) + (0.0524*P*)^2^] where *P* = (*F*_o_^2^ + 2*F*_c_^2^)/3
11092 reflections	(Δ/σ)_max_ = 0.002
715 parameters	Δρ_max_ = 0.86 e Å^−^^3^
6 restraints	Δρ_min_ = −0.68 e Å^−^^3^

### Special details

**Table:**

Geometry. All esds (except the esd in the dihedral angle between two l.s. planes) are estimated using the full covariance matrix. The cell esds are taken into account individually in the estimation of esds in distances, angles and torsion angles; correlations between esds in cell parameters are only used when they are defined by crystal symmetry. An approximate (isotropic) treatment of cell esds is used for estimating esds involving l.s. planes.
Refinement. Refinement of *F*^2^ against ALL reflections. The weighted *R*-factor wR and goodness of fit *S* are based on *F*^2^, conventional *R*-factors *R* are based on *F*, with *F* set to zero for negative *F*^2^. The threshold expression of *F*^2^ > σ(*F*^2^) is used only for calculating *R*-factors(gt) etc. and is not relevant to the choice of reflections for refinement. *R*-factors based on *F*^2^ are statistically about twice as large as those based on *F*, and *R*- factors based on ALL data will be even larger.

### Fractional atomic coordinates and isotropic or equivalent isotropic displacement parameters (Å^2^)

**Table:**

	*x*	*y*	*z*	*U*_iso_*/*U*_eq_	
Br1	0.43943 (3)	1.109304 (19)	0.27234 (2)	0.04887 (13)	
Br3	1.02769 (5)	0.78011 (2)	0.30945 (3)	0.07916 (19)	
Br4	0.95485 (4)	0.83143 (2)	0.11261 (3)	0.07435 (18)	
C1	0.7126 (4)	0.72823 (17)	0.15746 (18)	0.0462 (10)	
H1	0.7560	0.7213	0.1962	0.055*	
C2	0.7437 (4)	0.70462 (18)	0.1088 (2)	0.0532 (11)	
H2	0.8095	0.6812	0.1142	0.064*	
C3	0.6821 (4)	0.7139 (2)	0.0523 (2)	0.0566 (12)	
C4	0.5861 (4)	0.7474 (2)	0.0453 (2)	0.0592 (13)	
H4	0.5422	0.7536	0.0065	0.071*	
C5	0.5526 (4)	0.77185 (18)	0.09320 (19)	0.0499 (11)	
H5	0.4863	0.7948	0.0878	0.060*	
C6	0.6169 (3)	0.76234 (16)	0.14918 (17)	0.0395 (9)	
C7	0.7211 (5)	0.6888 (3)	−0.0003 (2)	0.0857 (18)	
H7A	0.7078	0.6424	−0.0042	0.129*	
H7B	0.7988	0.6972	0.0056	0.129*	
H7C	0.6818	0.7106	−0.0364	0.129*	
C8	0.5929 (3)	0.91727 (15)	0.23204 (15)	0.0294 (8)	
C9	0.5510 (3)	0.95961 (16)	0.18624 (15)	0.0289 (8)	
C10	0.5032 (3)	1.01623 (16)	0.19828 (16)	0.0320 (8)	
H10	0.4744	1.0456	0.1673	0.038*	
C11	0.4978 (3)	1.02969 (16)	0.25583 (17)	0.0336 (8)	
C12	0.5404 (3)	0.98801 (16)	0.30179 (16)	0.0327 (8)	
H12	0.5362	0.9981	0.3412	0.039*	
C13	0.5890 (3)	0.93175 (16)	0.29019 (16)	0.0315 (8)	
C14	0.4205 (3)	1.08761 (18)	0.03965 (16)	0.0368 (9)	
H14	0.4355	1.0557	0.0133	0.044*	
C15	0.3154 (3)	1.1000 (2)	0.04305 (18)	0.0469 (10)	
H15	0.2576	1.0777	0.0176	0.056*	
C16	0.2926 (4)	1.1446 (2)	0.0831 (2)	0.0539 (12)	
C17	0.3775 (4)	1.1780 (2)	0.11757 (19)	0.0545 (12)	
H17	0.3629	1.2091	0.1446	0.065*	
C18	0.4823 (4)	1.16789 (19)	0.11438 (17)	0.0478 (10)	
H18	0.5396	1.1918	0.1386	0.057*	
C19	0.5034 (3)	1.12220 (17)	0.07513 (16)	0.0329 (8)	
C20	0.1768 (4)	1.1552 (3)	0.0878 (3)	0.0891 (19)	
H20A	0.1673	1.1380	0.1256	0.134*	
H20B	0.1275	1.1331	0.0550	0.134*	
H20C	0.1607	1.2013	0.0857	0.134*	
C21	0.7461 (3)	1.01215 (16)	0.12285 (14)	0.0301 (8)	
C22	0.6975 (3)	0.95190 (17)	0.11682 (15)	0.0319 (8)	
C23	0.7602 (3)	0.89818 (17)	0.11292 (16)	0.0362 (9)	
H23	0.7281	0.8567	0.1080	0.043*	
C24	0.8693 (3)	0.90537 (17)	0.11629 (17)	0.0399 (9)	
C25	0.9191 (3)	0.96488 (17)	0.12485 (16)	0.0354 (9)	
H25	0.9948	0.9687	0.1280	0.043*	
C26	0.8572 (3)	1.01866 (16)	0.12870 (15)	0.0303 (8)	
C27	0.9046 (3)	1.10806 (16)	0.21337 (16)	0.0308 (8)	
C28	0.8342 (3)	1.15600 (16)	0.22221 (16)	0.0337 (8)	
H28	0.7958	1.1810	0.1899	0.040*	
C29	0.8207 (3)	1.16681 (17)	0.27889 (18)	0.0385 (9)	
C30	0.8731 (3)	1.13048 (17)	0.32628 (17)	0.0373 (9)	
H30	0.8619	1.1386	0.3647	0.045*	
C31	0.9428 (3)	1.08162 (16)	0.31765 (15)	0.0312 (8)	
C32	0.9604 (3)	1.07211 (15)	0.26131 (15)	0.0292 (8)	
C33	1.2307 (3)	0.99037 (18)	0.30119 (17)	0.0382 (9)	
C34	1.2480 (3)	0.9286 (2)	0.2833 (2)	0.0501 (11)	
H34	1.2192	0.9157	0.2435	0.060*	
C35	1.3071 (4)	0.8862 (2)	0.3234 (3)	0.0652 (14)	
H35	1.3184	0.8437	0.3111	0.078*	
C36	1.3498 (4)	0.9038 (3)	0.3804 (2)	0.0649 (14)	
C37	1.3352 (4)	0.9664 (3)	0.39852 (19)	0.0617 (13)	
H37	1.3666	0.9793	0.4381	0.074*	
C38	1.2746 (3)	1.0102 (2)	0.35870 (18)	0.0491 (10)	
H38	1.2636	1.0528	0.3708	0.059*	
C39	1.4138 (5)	0.8562 (3)	0.4245 (3)	0.0962 (19)	
H39A	1.3637	0.8277	0.4387	0.144*	
H39B	1.4584	0.8796	0.4581	0.144*	
H39C	1.4606	0.8306	0.4052	0.144*	
C40	0.9434 (3)	0.95975 (17)	0.36111 (15)	0.0347 (8)	
C41	1.0005 (3)	0.90881 (19)	0.34477 (17)	0.0423 (10)	
H41	1.0736	0.9143	0.3422	0.051*	
C42	0.9503 (4)	0.85022 (19)	0.33231 (18)	0.0475 (11)	
C43	0.8436 (3)	0.84099 (18)	0.33341 (17)	0.0423 (10)	
H43	0.8100	0.8004	0.3231	0.051*	
C44	0.7854 (3)	0.89146 (17)	0.34975 (16)	0.0350 (9)	
C45	0.8365 (3)	0.95058 (16)	0.36448 (15)	0.0330 (8)	
C46	0.8028 (3)	1.08666 (18)	0.46528 (16)	0.0371 (9)	
C47	0.7283 (3)	1.1358 (2)	0.44931 (18)	0.0463 (10)	
H47	0.6565	1.1267	0.4280	0.056*	
C48	0.7595 (4)	1.1981 (2)	0.46472 (19)	0.0510 (11)	
H48	0.7086	1.2320	0.4537	0.061*	
C49	0.8637 (4)	1.2122 (2)	0.49601 (19)	0.0519 (11)	
C50	0.9368 (4)	1.1623 (2)	0.51078 (18)	0.0512 (11)	
H50	1.0086	1.1715	0.5319	0.061*	
C51	0.9084 (3)	1.0994 (2)	0.49574 (17)	0.0451 (10)	
H51	0.9598	1.0656	0.5060	0.054*	
C52	0.8968 (4)	1.2802 (2)	0.5135 (2)	0.0706 (15)	
H52A	0.9342	1.2814	0.5555	0.106*	
H52B	0.8323	1.3076	0.5071	0.106*	
H52C	0.9454	1.2960	0.4894	0.106*	
C53	0.2434 (4)	0.9546 (2)	0.0855 (2)	0.0602 (12)	
H53A	0.3040	0.9636	0.0664	0.072*	
H53B	0.2442	0.9881	0.1160	0.072*	
Cl1	0.12058 (11)	0.95976 (7)	0.03170 (6)	0.0723 (4)	
Cl2	0.26337 (14)	0.88004 (7)	0.11968 (7)	0.0922 (5)	
O3	1.1686 (2)	1.10828 (12)	0.27040 (13)	0.0498 (7)	
O4	1.1667 (2)	1.02640 (14)	0.19338 (12)	0.0513 (7)	
O5	0.6387 (2)	1.07104 (15)	0.01904 (12)	0.0562 (8)	
O6	0.6901 (2)	1.16960 (14)	0.07755 (16)	0.0691 (10)	
O7	0.8372 (2)	0.96353 (13)	0.48243 (11)	0.0497 (7)	
O8	0.6506 (2)	1.00265 (14)	0.44481 (12)	0.0502 (7)	
O9	0.6457 (2)	0.86122 (10)	0.21968 (10)	0.0335 (6)	
O10	1.03130 (18)	1.02329 (11)	0.25253 (10)	0.0317 (5)	
O11	0.68364 (19)	1.06686 (11)	0.12749 (10)	0.0326 (6)	
O12	0.77790 (19)	1.00232 (11)	0.38031 (10)	0.0337 (6)	
S1	0.57961 (10)	0.79407 (4)	0.21118 (5)	0.0466 (3)	
S2	0.55608 (7)	0.94214 (4)	0.11169 (4)	0.0335 (2)	
S3	0.63585 (8)	1.10942 (5)	0.06895 (5)	0.0420 (2)	
S4	0.92238 (8)	1.09501 (4)	0.14036 (4)	0.0339 (2)	
S5	1.15421 (7)	1.04388 (4)	0.25031 (4)	0.0362 (2)	
S6	1.01038 (8)	1.03516 (5)	0.37934 (4)	0.0379 (2)	
S7	0.76318 (8)	1.00733 (5)	0.44783 (4)	0.0392 (2)	
S8	0.64672 (8)	0.88001 (4)	0.35001 (4)	0.0373 (2)	
Br2	0.72092 (4)	1.23092 (2)	0.29012 (2)	0.06032 (15)	
O1	0.4687 (3)	0.80924 (14)	0.19888 (16)	0.0672 (9)	
O2	0.6272 (3)	0.75471 (13)	0.26017 (13)	0.0712 (10)	

### Atomic displacement parameters (Å^2^)

**Table:**

	*U*^11^	*U*^22^	*U*^33^	*U*^12^	*U*^13^	*U*^23^
Br1	0.0508 (3)	0.0352 (2)	0.0657 (3)	0.01028 (19)	0.0236 (2)	−0.00070 (19)
Br3	0.0827 (4)	0.0475 (3)	0.1215 (5)	0.0245 (3)	0.0525 (4)	−0.0012 (3)
Br4	0.0645 (3)	0.0421 (3)	0.1291 (5)	0.0083 (2)	0.0485 (3)	−0.0082 (3)
C1	0.063 (3)	0.0278 (19)	0.042 (2)	−0.001 (2)	−0.001 (2)	−0.0005 (17)
C2	0.067 (3)	0.032 (2)	0.063 (3)	0.008 (2)	0.020 (2)	−0.005 (2)
C3	0.088 (4)	0.036 (2)	0.047 (3)	−0.004 (2)	0.018 (3)	−0.009 (2)
C4	0.088 (4)	0.039 (2)	0.042 (3)	−0.010 (2)	−0.002 (2)	−0.008 (2)
C5	0.055 (3)	0.032 (2)	0.056 (3)	−0.005 (2)	0.000 (2)	−0.002 (2)
C6	0.052 (3)	0.0226 (18)	0.044 (2)	−0.0067 (18)	0.0112 (19)	−0.0026 (16)
C7	0.137 (6)	0.071 (3)	0.058 (3)	0.007 (4)	0.039 (3)	−0.017 (3)
C8	0.0299 (19)	0.0239 (17)	0.035 (2)	−0.0016 (15)	0.0095 (16)	0.0010 (15)
C9	0.0266 (19)	0.0302 (18)	0.030 (2)	−0.0076 (15)	0.0079 (15)	−0.0013 (15)
C10	0.0252 (19)	0.0309 (19)	0.040 (2)	−0.0003 (15)	0.0080 (16)	0.0081 (16)
C11	0.029 (2)	0.0266 (18)	0.047 (2)	0.0013 (15)	0.0130 (17)	−0.0020 (16)
C12	0.032 (2)	0.0342 (19)	0.034 (2)	−0.0020 (16)	0.0124 (16)	−0.0034 (16)
C13	0.032 (2)	0.0303 (19)	0.034 (2)	−0.0035 (16)	0.0101 (16)	0.0032 (16)
C14	0.037 (2)	0.042 (2)	0.031 (2)	0.0025 (18)	0.0068 (17)	0.0034 (17)
C15	0.040 (2)	0.055 (3)	0.042 (2)	−0.002 (2)	0.0028 (19)	0.006 (2)
C16	0.049 (3)	0.064 (3)	0.054 (3)	0.017 (2)	0.023 (2)	0.020 (2)
C17	0.073 (3)	0.052 (3)	0.041 (3)	0.026 (2)	0.018 (2)	0.000 (2)
C18	0.057 (3)	0.044 (2)	0.038 (2)	0.007 (2)	0.002 (2)	−0.0011 (19)
C19	0.032 (2)	0.0336 (19)	0.032 (2)	0.0029 (16)	0.0068 (16)	0.0079 (16)
C20	0.060 (3)	0.109 (5)	0.108 (5)	0.034 (3)	0.040 (3)	0.035 (4)
C21	0.035 (2)	0.0316 (19)	0.0241 (19)	0.0019 (16)	0.0073 (15)	0.0024 (15)
C22	0.037 (2)	0.037 (2)	0.0225 (18)	−0.0049 (17)	0.0066 (15)	−0.0025 (15)
C23	0.045 (2)	0.0304 (19)	0.037 (2)	−0.0047 (17)	0.0159 (18)	−0.0014 (16)
C24	0.047 (2)	0.034 (2)	0.043 (2)	0.0083 (18)	0.0188 (19)	−0.0041 (17)
C25	0.035 (2)	0.040 (2)	0.034 (2)	0.0008 (17)	0.0138 (17)	−0.0012 (17)
C26	0.035 (2)	0.0304 (18)	0.0269 (19)	−0.0054 (16)	0.0096 (15)	−0.0014 (15)
C27	0.031 (2)	0.0266 (18)	0.036 (2)	−0.0074 (15)	0.0106 (16)	−0.0025 (15)
C28	0.034 (2)	0.0278 (18)	0.041 (2)	−0.0032 (16)	0.0124 (17)	0.0024 (16)
C29	0.033 (2)	0.0295 (19)	0.055 (3)	0.0014 (16)	0.0162 (19)	−0.0009 (18)
C30	0.039 (2)	0.036 (2)	0.039 (2)	−0.0001 (18)	0.0154 (18)	−0.0060 (17)
C31	0.030 (2)	0.0311 (18)	0.032 (2)	−0.0011 (16)	0.0057 (15)	−0.0013 (15)
C32	0.0292 (19)	0.0236 (17)	0.036 (2)	−0.0025 (15)	0.0094 (16)	−0.0028 (15)
C33	0.032 (2)	0.043 (2)	0.042 (2)	0.0081 (18)	0.0144 (17)	−0.0014 (18)
C34	0.049 (3)	0.047 (2)	0.058 (3)	0.005 (2)	0.020 (2)	0.000 (2)
C35	0.063 (3)	0.053 (3)	0.089 (4)	0.021 (2)	0.036 (3)	0.016 (3)
C36	0.054 (3)	0.079 (4)	0.072 (4)	0.028 (3)	0.036 (3)	0.033 (3)
C37	0.043 (3)	0.107 (4)	0.036 (3)	0.009 (3)	0.012 (2)	0.010 (3)
C38	0.041 (2)	0.062 (3)	0.046 (3)	0.005 (2)	0.014 (2)	−0.005 (2)
C39	0.091 (4)	0.111 (4)	0.093 (4)	0.030 (3)	0.035 (3)	0.044 (3)
C40	0.041 (2)	0.037 (2)	0.027 (2)	0.0070 (17)	0.0105 (16)	0.0078 (16)
C41	0.041 (2)	0.047 (2)	0.043 (2)	0.0112 (19)	0.0201 (19)	0.0078 (19)
C42	0.057 (3)	0.039 (2)	0.051 (3)	0.020 (2)	0.021 (2)	0.0051 (19)
C43	0.057 (3)	0.033 (2)	0.039 (2)	0.0083 (19)	0.016 (2)	0.0041 (17)
C44	0.041 (2)	0.034 (2)	0.031 (2)	0.0066 (17)	0.0105 (17)	0.0056 (16)
C45	0.041 (2)	0.0326 (19)	0.0262 (19)	0.0104 (17)	0.0091 (16)	0.0059 (15)
C46	0.040 (2)	0.041 (2)	0.033 (2)	0.0027 (18)	0.0123 (17)	−0.0044 (17)
C47	0.040 (2)	0.052 (3)	0.048 (3)	0.005 (2)	0.0128 (19)	−0.008 (2)
C48	0.053 (3)	0.047 (2)	0.057 (3)	0.009 (2)	0.022 (2)	−0.005 (2)
C49	0.064 (3)	0.052 (3)	0.048 (3)	−0.008 (2)	0.029 (2)	−0.014 (2)
C50	0.043 (3)	0.069 (3)	0.042 (3)	−0.008 (2)	0.010 (2)	−0.011 (2)
C51	0.050 (3)	0.050 (2)	0.034 (2)	0.006 (2)	0.0080 (19)	−0.0065 (19)
C52	0.084 (4)	0.057 (3)	0.077 (4)	−0.018 (3)	0.032 (3)	−0.023 (3)
C53	0.063 (3)	0.071 (3)	0.054 (3)	0.004 (3)	0.028 (2)	−0.005 (2)
Cl1	0.0642 (8)	0.0817 (9)	0.0734 (9)	0.0079 (7)	0.0210 (7)	0.0129 (7)
Cl2	0.1032 (12)	0.0880 (10)	0.0814 (11)	0.0135 (9)	0.0135 (8)	0.0221 (8)
O3	0.0345 (16)	0.0359 (15)	0.079 (2)	−0.0035 (12)	0.0133 (14)	−0.0007 (14)
O4	0.0414 (17)	0.076 (2)	0.0404 (16)	0.0152 (15)	0.0182 (13)	0.0060 (14)
O5	0.0519 (18)	0.087 (2)	0.0340 (16)	0.0226 (16)	0.0194 (13)	0.0147 (15)
O6	0.0445 (19)	0.0490 (18)	0.108 (3)	−0.0096 (15)	0.0045 (17)	0.0377 (18)
O7	0.064 (2)	0.0514 (17)	0.0335 (16)	0.0069 (15)	0.0114 (13)	0.0064 (13)
O8	0.0486 (18)	0.0607 (18)	0.0476 (17)	−0.0062 (15)	0.0242 (14)	−0.0130 (14)
O9	0.0419 (15)	0.0242 (12)	0.0362 (14)	−0.0009 (11)	0.0124 (12)	−0.0009 (10)
O10	0.0299 (13)	0.0293 (13)	0.0365 (14)	0.0017 (10)	0.0088 (11)	−0.0030 (10)
O11	0.0349 (14)	0.0299 (13)	0.0316 (14)	0.0035 (11)	0.0047 (11)	0.0047 (10)
O12	0.0375 (15)	0.0351 (13)	0.0298 (14)	0.0077 (11)	0.0106 (11)	0.0000 (11)
S1	0.0672 (8)	0.0267 (5)	0.0518 (7)	−0.0111 (5)	0.0257 (6)	−0.0016 (4)
S2	0.0339 (5)	0.0351 (5)	0.0306 (5)	−0.0054 (4)	0.0057 (4)	0.0000 (4)
S3	0.0364 (6)	0.0448 (6)	0.0452 (6)	0.0024 (5)	0.0104 (5)	0.0167 (5)
S4	0.0348 (5)	0.0349 (5)	0.0333 (5)	−0.0059 (4)	0.0106 (4)	0.0016 (4)
S5	0.0310 (5)	0.0398 (5)	0.0397 (6)	0.0021 (4)	0.0124 (4)	0.0005 (4)
S6	0.0362 (5)	0.0461 (5)	0.0315 (5)	0.0035 (4)	0.0082 (4)	0.0009 (4)
S7	0.0444 (6)	0.0439 (5)	0.0321 (5)	0.0015 (5)	0.0146 (4)	−0.0026 (4)
S8	0.0449 (6)	0.0352 (5)	0.0335 (5)	0.0013 (4)	0.0127 (4)	0.0074 (4)
Br2	0.0705 (3)	0.0490 (3)	0.0703 (3)	0.0274 (2)	0.0344 (3)	0.0062 (2)
O1	0.058 (2)	0.0509 (18)	0.103 (3)	−0.0244 (16)	0.0409 (19)	−0.0173 (18)
O2	0.140 (3)	0.0304 (15)	0.049 (2)	−0.0058 (18)	0.034 (2)	0.0075 (14)

### Geometric parameters (Å, °)

**Table:**

Br1—C11	1.888 (3)	C30—C31	1.391 (5)
Br3—C42	1.900 (4)	C30—H30	0.9500
Br4—C24	1.896 (4)	C31—C32	1.389 (5)
C1—C2	1.370 (6)	C31—S6	1.775 (4)
C1—C6	1.381 (5)	C32—O10	1.403 (4)
C1—H1	0.9500	C33—C34	1.382 (5)
C2—C3	1.376 (6)	C33—C38	1.384 (5)
C2—H2	0.9500	C33—S5	1.745 (4)
C3—C4	1.380 (7)	C34—C35	1.373 (6)
C3—C7	1.510 (6)	C34—H34	0.9500
C4—C5	1.377 (6)	C35—C36	1.357 (7)
C4—H4	0.9500	C35—H35	0.9500
C5—C6	1.379 (6)	C36—C37	1.394 (7)
C5—H5	0.9500	C36—C39	1.518 (7)
C6—S1	1.744 (4)	C37—C38	1.395 (6)
C7—H7A	0.9800	C37—H37	0.9500
C7—H7B	0.9800	C38—H38	0.9500
C7—H7C	0.9800	C39—H39A	0.9800
C8—C9	1.387 (5)	C39—H39B	0.9800
C8—C13	1.394 (5)	C39—H39C	0.9800
C8—O9	1.407 (4)	C40—C41	1.385 (5)
C9—C10	1.382 (5)	C40—C45	1.391 (5)
C9—S2	1.782 (3)	C40—S6	1.787 (4)
C10—C11	1.381 (5)	C41—C42	1.374 (6)
C10—H10	0.9500	C41—H41	0.9500
C11—C12	1.383 (5)	C42—C43	1.375 (6)
C12—C13	1.377 (5)	C43—C44	1.386 (5)
C12—H12	0.9500	C43—H43	0.9500
C13—S8	1.775 (4)	C44—C45	1.395 (5)
C14—C15	1.378 (5)	C44—S8	1.778 (4)
C14—C19	1.379 (5)	C45—O12	1.404 (4)
C14—H14	0.9500	C46—C47	1.383 (5)
C15—C16	1.389 (6)	C46—C51	1.389 (6)
C15—H15	0.9500	C46—S7	1.744 (4)
C16—C17	1.373 (6)	C47—C48	1.378 (6)
C16—C20	1.515 (6)	C47—H47	0.9500
C17—C18	1.365 (6)	C48—C49	1.385 (6)
C17—H17	0.9500	C48—H48	0.9500
C18—C19	1.384 (5)	C49—C50	1.383 (6)
C18—H18	0.9500	C49—C52	1.504 (6)
C19—S3	1.741 (4)	C50—C51	1.380 (6)
C20—H20A	0.9800	C50—H50	0.9500
C20—H20B	0.9800	C51—H51	0.9500
C20—H20C	0.9800	C52—H52A	0.9800
C21—C22	1.389 (5)	C52—H52B	0.9800
C21—C26	1.391 (5)	C52—H52C	0.9800
C21—O11	1.405 (4)	C53—Cl2	1.734 (5)
C22—C23	1.387 (5)	C53—Cl1	1.760 (5)
C22—S2	1.783 (4)	C53—H53A	0.9900
C23—C24	1.378 (5)	C53—H53B	0.9900
C23—H23	0.9500	O3—S5	1.416 (3)
C24—C25	1.383 (5)	O4—S5	1.413 (3)
C25—C26	1.382 (5)	O5—S3	1.413 (3)
C25—H25	0.9500	O6—S3	1.420 (3)
C26—S4	1.782 (3)	O7—S7	1.417 (3)
C27—C28	1.385 (5)	O8—S7	1.418 (3)
C27—C32	1.390 (5)	O9—S1	1.618 (2)
C27—S4	1.780 (4)	O10—S5	1.630 (2)
C28—C29	1.383 (5)	O11—S3	1.617 (2)
C28—H28	0.9500	O12—S7	1.623 (2)
C29—C30	1.372 (5)	S1—O1	1.407 (3)
C29—Br2	1.898 (3)	S1—O2	1.417 (3)
			
C2—C1—C6	118.8 (4)	C34—C33—S5	119.4 (3)
C2—C1—H1	120.6	C38—C33—S5	119.9 (3)
C6—C1—H1	120.6	C35—C34—C33	119.5 (4)
C1—C2—C3	121.7 (4)	C35—C34—H34	120.2
C1—C2—H2	119.1	C33—C34—H34	120.2
C3—C2—H2	119.1	C36—C35—C34	121.3 (5)
C2—C3—C4	118.4 (4)	C36—C35—H35	119.3
C2—C3—C7	120.0 (5)	C34—C35—H35	119.3
C4—C3—C7	121.6 (5)	C35—C36—C37	119.7 (4)
C5—C4—C3	121.4 (4)	C35—C36—C39	120.8 (5)
C5—C4—H4	119.3	C37—C36—C39	119.5 (5)
C3—C4—H4	119.3	C36—C37—C38	120.1 (4)
C4—C5—C6	118.8 (4)	C36—C37—H37	120.0
C4—C5—H5	120.6	C38—C37—H37	120.0
C6—C5—H5	120.6	C33—C38—C37	118.8 (4)
C5—C6—C1	121.0 (4)	C33—C38—H38	120.6
C5—C6—S1	120.5 (3)	C37—C38—H38	120.6
C1—C6—S1	118.5 (3)	C36—C39—H39A	109.5
C3—C7—H7A	109.5	C36—C39—H39B	109.5
C3—C7—H7B	109.5	H39A—C39—H39B	109.5
H7A—C7—H7B	109.5	C36—C39—H39C	109.5
C3—C7—H7C	109.5	H39A—C39—H39C	109.5
H7A—C7—H7C	109.5	H39B—C39—H39C	109.5
H7B—C7—H7C	109.5	C41—C40—C45	119.3 (4)
C9—C8—C13	120.7 (3)	C41—C40—S6	119.0 (3)
C9—C8—O9	119.1 (3)	C45—C40—S6	121.6 (3)
C13—C8—O9	120.1 (3)	C42—C41—C40	119.3 (4)
C10—C9—C8	119.6 (3)	C42—C41—H41	120.4
C10—C9—S2	119.0 (3)	C40—C41—H41	120.4
C8—C9—S2	121.3 (3)	C41—C42—C43	122.1 (3)
C11—C10—C9	119.4 (3)	C41—C42—Br3	119.6 (3)
C11—C10—H10	120.3	C43—C42—Br3	118.2 (3)
C9—C10—H10	120.3	C42—C43—C44	119.3 (4)
C10—C11—C12	121.3 (3)	C42—C43—H43	120.3
C10—C11—Br1	118.9 (3)	C44—C43—H43	120.3
C12—C11—Br1	119.7 (3)	C43—C44—C45	119.2 (3)
C13—C12—C11	119.7 (3)	C43—C44—S8	119.4 (3)
C13—C12—H12	120.2	C45—C44—S8	121.5 (3)
C11—C12—H12	120.2	C40—C45—C44	120.8 (3)
C12—C13—C8	119.3 (3)	C40—C45—O12	119.5 (3)
C12—C13—S8	119.0 (3)	C44—C45—O12	119.6 (3)
C8—C13—S8	121.7 (3)	C47—C46—C51	121.1 (4)
C15—C14—C19	118.9 (4)	C47—C46—S7	119.6 (3)
C15—C14—H14	120.5	C51—C46—S7	119.4 (3)
C19—C14—H14	120.5	C48—C47—C46	119.2 (4)
C14—C15—C16	121.1 (4)	C48—C47—H47	120.4
C14—C15—H15	119.4	C46—C47—H47	120.4
C16—C15—H15	119.4	C47—C48—C49	121.2 (4)
C17—C16—C15	118.0 (4)	C47—C48—H48	119.4
C17—C16—C20	122.0 (5)	C49—C48—H48	119.4
C15—C16—C20	120.0 (5)	C50—C49—C48	118.4 (4)
C18—C17—C16	122.3 (4)	C50—C49—C52	120.6 (4)
C18—C17—H17	118.8	C48—C49—C52	121.0 (4)
C16—C17—H17	118.8	C51—C50—C49	121.9 (4)
C17—C18—C19	118.7 (4)	C51—C50—H50	119.0
C17—C18—H18	120.7	C49—C50—H50	119.0
C19—C18—H18	120.7	C50—C51—C46	118.3 (4)
C14—C19—C18	120.9 (4)	C50—C51—H51	120.9
C14—C19—S3	119.3 (3)	C46—C51—H51	120.9
C18—C19—S3	119.7 (3)	C49—C52—H52A	109.5
C16—C20—H20A	109.5	C49—C52—H52B	109.5
C16—C20—H20B	109.5	H52A—C52—H52B	109.5
H20A—C20—H20B	109.5	C49—C52—H52C	109.5
C16—C20—H20C	109.5	H52A—C52—H52C	109.5
H20A—C20—H20C	109.5	H52B—C52—H52C	109.5
H20B—C20—H20C	109.5	Cl2—C53—Cl1	113.0 (3)
C22—C21—C26	121.0 (3)	Cl2—C53—H53A	109.0
C22—C21—O11	119.5 (3)	Cl1—C53—H53A	109.0
C26—C21—O11	119.4 (3)	Cl2—C53—H53B	109.0
C23—C22—C21	119.0 (3)	Cl1—C53—H53B	109.0
C23—C22—S2	119.2 (3)	H53A—C53—H53B	107.8
C21—C22—S2	121.8 (3)	C8—O9—S1	118.8 (2)
C24—C23—C22	119.6 (3)	C32—O10—S5	117.8 (2)
C24—C23—H23	120.2	C21—O11—S3	119.2 (2)
C22—C23—H23	120.2	C45—O12—S7	119.2 (2)
C23—C24—C25	121.7 (3)	O1—S1—O2	120.9 (2)
C23—C24—Br4	119.2 (3)	O1—S1—O9	107.33 (15)
C25—C24—Br4	119.0 (3)	O2—S1—O9	106.71 (17)
C26—C25—C24	119.0 (3)	O1—S1—C6	111.5 (2)
C26—C25—H25	120.5	O2—S1—C6	107.20 (19)
C24—C25—H25	120.5	O9—S1—C6	101.25 (15)
C25—C26—C21	119.6 (3)	C9—S2—C22	99.98 (16)
C25—C26—S4	118.6 (3)	O5—S3—O6	120.6 (2)
C21—C26—S4	121.8 (3)	O5—S3—O11	107.63 (15)
C28—C27—C32	119.7 (3)	O6—S3—O11	106.77 (17)
C28—C27—S4	118.6 (3)	O5—S3—C19	111.19 (18)
C32—C27—S4	121.7 (3)	O6—S3—C19	107.50 (18)
C29—C28—C27	118.9 (3)	O11—S3—C19	101.35 (15)
C29—C28—H28	120.5	C27—S4—C26	97.10 (15)
C27—C28—H28	120.5	O4—S5—O3	121.12 (18)
C30—C29—C28	121.9 (3)	O4—S5—O10	106.07 (15)
C30—C29—Br2	119.5 (3)	O3—S5—O10	106.85 (14)
C28—C29—Br2	118.5 (3)	O4—S5—C33	107.62 (17)
C29—C30—C31	119.6 (3)	O3—S5—C33	111.79 (19)
C29—C30—H30	120.2	O10—S5—C33	101.43 (15)
C31—C30—H30	120.2	C31—S6—C40	99.90 (17)
C32—C31—C30	119.0 (3)	O7—S7—O8	120.84 (18)
C32—C31—S6	121.9 (3)	O7—S7—O12	107.23 (14)
C30—C31—S6	119.1 (3)	O8—S7—O12	106.70 (15)
C31—C32—C27	120.8 (3)	O7—S7—C46	111.01 (18)
C31—C32—O10	119.2 (3)	O8—S7—C46	108.04 (17)
C27—C32—O10	119.9 (3)	O12—S7—C46	101.15 (15)
C34—C33—C38	120.6 (4)	C13—S8—C44	98.43 (16)
			
C6—C1—C2—C3	−0.1 (6)	C41—C42—C43—C44	−2.5 (6)
C1—C2—C3—C4	−0.8 (7)	Br3—C42—C43—C44	−179.8 (3)
C1—C2—C3—C7	178.1 (4)	C42—C43—C44—C45	0.3 (5)
C2—C3—C4—C5	0.9 (7)	C42—C43—C44—S8	179.0 (3)
C7—C3—C4—C5	−178.0 (4)	C41—C40—C45—C44	−2.1 (5)
C3—C4—C5—C6	−0.1 (6)	S6—C40—C45—C44	179.6 (3)
C4—C5—C6—C1	−0.9 (6)	C41—C40—C45—O12	−179.2 (3)
C4—C5—C6—S1	178.4 (3)	S6—C40—C45—O12	2.5 (4)
C2—C1—C6—C5	1.0 (6)	C43—C44—C45—C40	2.0 (5)
C2—C1—C6—S1	−178.3 (3)	S8—C44—C45—C40	−176.7 (3)
C13—C8—C9—C10	−1.2 (5)	C43—C44—C45—O12	179.1 (3)
O9—C8—C9—C10	−177.1 (3)	S8—C44—C45—O12	0.4 (5)
C13—C8—C9—S2	179.6 (3)	C51—C46—C47—C48	−0.9 (6)
O9—C8—C9—S2	3.6 (4)	S7—C46—C47—C48	178.2 (3)
C8—C9—C10—C11	−0.1 (5)	C46—C47—C48—C49	−0.3 (6)
S2—C9—C10—C11	179.2 (3)	C47—C48—C49—C50	1.0 (6)
C9—C10—C11—C12	0.8 (5)	C47—C48—C49—C52	−179.0 (4)
C9—C10—C11—Br1	176.6 (3)	C48—C49—C50—C51	−0.6 (6)
C10—C11—C12—C13	−0.3 (5)	C52—C49—C50—C51	179.4 (4)
Br1—C11—C12—C13	−176.0 (3)	C49—C50—C51—C46	−0.5 (6)
C11—C12—C13—C8	−1.0 (5)	C47—C46—C51—C50	1.3 (6)
C11—C12—C13—S8	178.2 (3)	S7—C46—C51—C50	−177.8 (3)
C9—C8—C13—C12	1.7 (5)	C9—C8—O9—S1	−99.3 (3)
O9—C8—C13—C12	177.6 (3)	C13—C8—O9—S1	84.7 (4)
C9—C8—C13—S8	−177.4 (3)	C31—C32—O10—S5	−99.5 (3)
O9—C8—C13—S8	−1.5 (4)	C27—C32—O10—S5	83.7 (3)
C19—C14—C15—C16	−2.7 (6)	C22—C21—O11—S3	−97.4 (3)
C14—C15—C16—C17	2.6 (6)	C26—C21—O11—S3	87.3 (3)
C14—C15—C16—C20	−177.3 (4)	C40—C45—O12—S7	−100.4 (3)
C15—C16—C17—C18	−1.1 (6)	C44—C45—O12—S7	82.5 (4)
C20—C16—C17—C18	178.8 (4)	C8—O9—S1—O1	19.2 (3)
C16—C17—C18—C19	−0.3 (6)	C8—O9—S1—O2	−111.8 (3)
C15—C14—C19—C18	1.2 (5)	C8—O9—S1—C6	136.2 (3)
C15—C14—C19—S3	−177.2 (3)	C5—C6—S1—O1	19.2 (4)
C17—C18—C19—C14	0.3 (6)	C1—C6—S1—O1	−161.5 (3)
C17—C18—C19—S3	178.7 (3)	C5—C6—S1—O2	153.7 (3)
C26—C21—C22—C23	−4.1 (5)	C1—C6—S1—O2	−27.0 (4)
O11—C21—C22—C23	−179.3 (3)	C5—C6—S1—O9	−94.7 (3)
C26—C21—C22—S2	177.9 (2)	C1—C6—S1—O9	84.6 (3)
O11—C21—C22—S2	2.7 (4)	C10—C9—S2—C22	109.5 (3)
C21—C22—C23—C24	1.3 (5)	C8—C9—S2—C22	−71.2 (3)
S2—C22—C23—C24	179.4 (3)	C23—C22—S2—C9	109.9 (3)
C22—C23—C24—C25	1.5 (6)	C21—C22—S2—C9	−72.1 (3)
C22—C23—C24—Br4	178.7 (3)	C21—O11—S3—O5	18.4 (3)
C23—C24—C25—C26	−1.6 (6)	C21—O11—S3—O6	−112.4 (3)
Br4—C24—C25—C26	−178.8 (3)	C21—O11—S3—C19	135.2 (3)
C24—C25—C26—C21	−1.2 (5)	C14—C19—S3—O5	10.2 (3)
C24—C25—C26—S4	179.5 (3)	C18—C19—S3—O5	−168.2 (3)
C22—C21—C26—C25	4.0 (5)	C14—C19—S3—O6	144.2 (3)
O11—C21—C26—C25	179.2 (3)	C18—C19—S3—O6	−34.2 (4)
C22—C21—C26—S4	−176.7 (3)	C14—C19—S3—O11	−103.9 (3)
O11—C21—C26—S4	−1.4 (4)	C18—C19—S3—O11	77.7 (3)
C32—C27—C28—C29	−0.3 (5)	C28—C27—S4—C26	−110.9 (3)
S4—C27—C28—C29	−179.4 (3)	C32—C27—S4—C26	69.9 (3)
C27—C28—C29—C30	−1.5 (5)	C25—C26—S4—C27	−111.9 (3)
C27—C28—C29—Br2	−178.2 (3)	C21—C26—S4—C27	68.8 (3)
C28—C29—C30—C31	0.6 (6)	C32—O10—S5—O4	−118.6 (2)
Br2—C29—C30—C31	177.3 (3)	C32—O10—S5—O3	11.9 (3)
C29—C30—C31—C32	2.1 (5)	C32—O10—S5—C33	129.1 (2)
C29—C30—C31—S6	179.8 (3)	C34—C33—S5—O4	−28.7 (4)
C30—C31—C32—C27	−3.8 (5)	C38—C33—S5—O4	150.4 (3)
S6—C31—C32—C27	178.5 (3)	C34—C33—S5—O3	−164.0 (3)
C30—C31—C32—O10	179.4 (3)	C38—C33—S5—O3	15.1 (4)
S6—C31—C32—O10	1.7 (4)	C34—C33—S5—O10	82.5 (3)
C28—C27—C32—C31	3.0 (5)	C38—C33—S5—O10	−98.4 (3)
S4—C27—C32—C31	−177.9 (3)	C32—C31—S6—C40	−71.4 (3)
C28—C27—C32—O10	179.7 (3)	C30—C31—S6—C40	110.9 (3)
S4—C27—C32—O10	−1.2 (4)	C41—C40—S6—C31	110.3 (3)
C38—C33—C34—C35	1.6 (6)	C45—C40—S6—C31	−71.5 (3)
S5—C33—C34—C35	−179.3 (3)	C45—O12—S7—O7	13.5 (3)
C33—C34—C35—C36	−0.5 (7)	C45—O12—S7—O8	−117.3 (3)
C34—C35—C36—C37	−1.1 (7)	C45—O12—S7—C46	129.9 (3)
C34—C35—C36—C39	179.2 (4)	C47—C46—S7—O7	−162.0 (3)
C35—C36—C37—C38	1.7 (7)	C51—C46—S7—O7	17.1 (4)
C39—C36—C37—C38	−178.6 (4)	C47—C46—S7—O8	−27.4 (3)
C34—C33—C38—C37	−1.0 (6)	C51—C46—S7—O8	151.7 (3)
S5—C33—C38—C37	179.9 (3)	C47—C46—S7—O12	84.5 (3)
C36—C37—C38—C33	−0.6 (6)	C51—C46—S7—O12	−96.4 (3)
C45—C40—C41—C42	−0.1 (5)	C12—C13—S8—C44	−110.6 (3)
S6—C40—C41—C42	178.2 (3)	C8—C13—S8—C44	68.6 (3)
C40—C41—C42—C43	2.4 (6)	C43—C44—S8—C13	−112.0 (3)
C40—C41—C42—Br3	179.7 (3)	C45—C44—S8—C13	66.6 (3)

### Hydrogen-bond geometry (Å, °)

**Table:**

*C*g1, *C*g2, *C*g3 and *C*g4 are the centroids of the C14–C19, C1–C6, C51–C56 and C33–C38 rings, respectively.

**Table:**

*D*—H···*A*	*D*—H	H···*A*	*D*···*A*	*D*—H···*A*
C12—H12···O8	0.95	2.51	3.303 (5)	141
C53—H53A···O5^i^	0.99	2.38	3.175 (5)	137
C53—H53B···O4^ii^	0.99	2.37	3.249 (5)	147
C17—H17···O2^iii^	0.95	2.38	3.266 (5)	155
C10—H10···Cg1	0.95	2.75	3.698 (1)	178
C23—H23···Cg2	0.95	2.65	3.602 (1)	176
C30—H30···Cg3	0.95	2.80	3.742 (1)	174
C41—H41···Cg4	0.95	2.85	3.794 (1)	172

Symmetry codes: (i) −*x*+1, −*y*+2, −*z*; (ii) *x*−1, *y*, *z*; (iii) −*x*+1, *y*+1/2, −*z*+1/2.
